# High Melt Strength Recycled High-Density Polyethylene: Evaluation of a Novel Route for Targeting the Polymer Microstructure

**DOI:** 10.3390/polym17030382

**Published:** 2025-01-30

**Authors:** Giulia Bernagozzi, Rossella Arrigo, Alberto Frache

**Affiliations:** 1 Department of Applied Science and Technology, Politecnico di Torino, Viale Teresa Michel 5, 15121 Alessandria, Italy; giulia.bernagozzi@polito.it (G.B.); alberto.frache@polito.it (A.F.); 2 Local INSTM Unit, 15121 Alessandria, Italy

**Keywords:** HDPE, melt strength, mechanical recycling, plastic circularity, upcycling

## Abstract

The mechanical recycling of thermoplastics (especially of polyolefins) often results in recyclates with inferior properties compared to their virgin counterparts. This phenomenon is mainly due to the modification of the polymer microstructure induced by the degradation processes undergone by the materials during their service life and reprocessing. In this work, a promising route for obtaining high-melt-strength recycled high-density polyethylene (HDPE) is proposed. In particular, the exploited approach involves the utilization of a commercially available additive (i.e., Nexamite^®^ R305, Nexam Chemical, Lomma, Sweden), which was demonstrated to be capable of driving thermo-mechanical degradation reactions (experienced by HDPE during mechanical recycling) towards the obtainment of a long-chain branched microstructure, thereby enabling the further processing of the recycled material through technologies dominated by elongational flow. The additive-induced alterations of the polymer microstructure were exploited for the formulation of fibers, and the performed tensile characterization showed that the additive-containing material exhibits strikingly improved ductility (namely, elongation at break of 350% for the fibers stretched at a draw ratio of 60) with respect to pristine recycled HDPE. Overall, the obtained results clearly demonstrated the possibility of attaining an effective upcycling of HDPE, which could be exploited for industrially relevant high-added-value applications, hence paving the way for the achievement of full plastic circularity.

## 1. Introduction

According to the latest available data [1], high-density polyethylene (HDPE) is one of the most used thermoplastic polymers, at least as far as the European market is concerned, esteemed for its chemical resistance, mechanical strength, and versatility. Its widespread applications in industries ranging from packaging and construction to automotive and healthcare have led to a significant increase in its production over recent decades [2]. However, the extensive utilization of HDPE has also prompted growing concerns regarding environmental sustainability, particularly the management of its end-of-life products [3,4]. The mechanical recycling of HDPE undoubtedly offers a promising avenue to mitigate the environmental impact associated with its disposal while conserving valuable resources [2,5,6,7]. Nevertheless, achieving efficient recycling processes requires a comprehensive understanding of HDPE’s degradation mechanism, which can significantly affect the physical and mechanical properties of the polymer, as well as its processability, stability, and recyclability [7]. In fact, in most cases, recycled thermoplastics (and polyolefins, specifically) possess a lower quality compared to their virgin counterparts, forcing their final use towards applications with low-engineering requirements [8]. As a matter of fact, the reuse of recycled HDPE in the same application as virgin polymers, ensuring, at the same time, unmodified performance, often requires the introduction of a certain amount of virgin HDPE in the blend. For example, it has been shown that, for the formulation of bottles for cleaning products, high levels of environmental stress cracking resistance are mandatory. However, due to the modifications of the polymer microstructure induced by degradation, this property is typically inferior in post-consumer recycled plastics, thus limiting their effective exploitation in the rigid-packaging industry [9]. Similarly, recycled HDPE recovered from pressure pipes is not currently utilized for the same applications, mainly due to the high structural and loading requirements (in terms of slow crack growth and rapid crack propagation resistances) that must be guaranteed [10]. All these issues ultimately compromise the achievement of full plastic circularity; therefore, the obtainment of HDPE recyclates with high added value and performances at least comparable to those of virgin materials is mandatory for the future of recycled polyolefins.

As widely reported in the literature [11,12,13,14,15,16], the thermo-mechanical degradation of HDPE, typically experienced by the polymer during mechanical recycling processes, involves the concurrent occurrence of different phenomena, such as chain scission reactions, branching formation, and crosslinking, resulting in a remarkable alteration of the macromolecular architecture of the polymer chains. Furthermore, it has been demonstrated that the extent of each phenomenon depends on several factors, such as the catalyst type employed for the polymerization process [15] and the type and concentration of unsaturated groups, as well as on the processing conditions exploited for the reprocessing [11,12,14,16]. From a general point of view, during the reprocessing of HDPE, the degradation mechanism starts with chain scission reactions, followed by the recombination of the formed radicals. Therefore, when macroradicals react with the vinyl terminal unsaturation of other chains, the formation of short- and long-chain branches occurs [15]. In most cases, when likely chain scission, branching, and crosslinking reactions occur simultaneously, the mean melt flow rate (MFR) value decreases [13,17,18,19,20] and the viscosity increases [14,20,21,22], indicating a reduction in the macromolecular mobility due to chain branching and crosslinking. Since the reported increase in viscosity and the modified macromolecular architecture can lead to the unsuccessful reprocessing of HDPE, the adjustment of process settings is typically required when dealing with this kind of recyclates [18]. In addition, it is worth pointing out that the final mechanical properties of recycled HDPE are usually poorer than the virgin polymer; once again, this can be ascribed to the heterogeneous microstructure characterizing the recycled material [14,23].

Therefore, in order to effectively implement a circular economy model which aims to reuse and recycle the plastic products already in circulation, the microstructural modification induced by degradation should be somehow controlled, to drive it towards a specific microstructure which allows recycled HDPE to be used in valuable applications or, at least, in the same initial use cases.

A very effective method to modify HDPE processability is the introduction of long-chain branches (LCB) on the main chain backbone [24,25,26,27]. In this context, Cheng et al. [24] proved that irradiation with an electron-beam in air at low doses of HDPE can promote the formation of LCB, enhancing the processability in terms of sag resistance and strain hardening. Furthermore, the obtainment of increased values of melt strength was also demonstrated. Aiming to find a method for making HDPE and polypropylene waste compatible, Wei et al. [27] introduced ozone-to-oxidize HDPE and a multifunctional monomer to promote chain branching, with a consequent enhancement in the rheological and mechanical properties. The formation of long-chain branching can also be successfully obtained through the addition of peroxides both on virgin [25] and recycled HDPE [26]. Furthermore, the reactive extrusion of recycled HDPE with various contents of peroxides, aiming to introduce LCB, results in increased melt strength and extensional viscosity and the appearance of a strain-hardening behavior [28].

Therefore, the presence of LCB induces a significant modification of the processability of these products, allowing the employment of polymers in several industrially relevant processing technologies (such as fiber spinning, film blowing, blow molding, and foaming) dominated by elongational flow [29,30,31]. In the context of mechanical recycling, the introduction of LCBs on a recycled polymer could open new perspectives towards closed-loop recycling strategies or upcycling approaches, resulting in high-added-value products that can be employed in applications with high-engineering requirements.

Keeping this in mind, in the present work, an effective and industrially viable strategy for obtaining recycled HDPE with a high melt strength, and hence improved processability upon elongational flow, was proposed. To this aim, a commercially available additive (Nexamite^®^ R305, NEX) was introduced in a degraded HDPE sample, and the so-obtained material was subjected to further reprocessing. The evolution of the macromolecular architecture of the polymer was monitored through rheological and spectroscopic analyses, demonstrating that NEX is able to drive the degradation reactions occurring during reprocessing towards the formation of long-chain branching structures. It is important to highlight that the use of additives for modifying the macromolecular microstructure of recycled polyolefins is not a commonly exploited route. In fact, differently from other thermoplastics, such as polyesters, for which the utilization of chain extenders is a typical method allowing one to restore the initial molecular weight of the virgin polymer [32], for polyolefins, the sole route currently proposed is the introduction of peroxides that, however, bring about to the non-selective formation of crosslinks [33,34].

Then, the melt strength of the recycled HDPE (with and without the additive) was evaluated, and the materials were subjected to non-isothermal elongational flow for obtaining fibers at different draw ratios, which were further characterized through tensile tests. Overall, the obtained results clearly demonstrate that recycled HDPE having the proper processability for elongational flow-dominated technologies can be easily obtained, opening new avenues for the formulation of high-added-value recyclates.

## 2. Materials and Methods

### 2.1. Materials

The materials used in this work were the following:

- High-density polyethylene (HDPE) Eraclene MS80U was supplied by Versalis (San Donato Milanese (MI), Italy), having a melt flow rate of 27 g/10 min (190 °C/2.16 kg) and a density of 0.955 g/cm^3^. According to the datasheet specifications, this HDPE was characterized by a narrow molecular weight distribution, making this HDPE ideal for injection molding applications.
- Nexamite^®^ R305 (NEX) was supplied by Nexam Chemical (Lomma, Sweden). This additive was a polyethylene-based silane technology (peroxide free) with a melt flow rate of 1.0 g/10min (190 °C/2.16 kg).

### 2.2. Processing

Melt processing was carried out by means of a twin-screw extruder Process 11 (Thermo Fisher Scientific, Waltham, MA, USA) equipped with 11 mm diameter screws (L/D = 40) and a screw profile with an alternance of conveying and kneading screw elements. The used processing parameters were the following: barrel temperature 190 °C, screw speed 250 rpm, and feed rate 800 g/h.

The virgin HDPE pellets, which did not experience any processing, were named HDPE_V. This sample was subjected to 30 subsequent extrusion cycles, until reaching a high extent of degradation, and the so-obtained material was named HDPE_D. Subsequently, HDPE_D was further processed for 1 min or 5 min without (HDPE_R1 and HDPE_R5, respectively) and with 5 wt.% of the additive (HDPE_RN1 and HDPE_RN5, respectively).

In addition, HDPE_R5 and HDPE_RN5 fibers were produced using a Rheospin apparatus (IdeaInstr, Italy), already described in a previous work [35]. Briefly, this apparatus is equipped with a series of pulleys that collect the hot filament coming from the extruder and transfer it to a final pulley rotating at a constant speed. In this way, the filament is subjected to a non-isothermal elongation flow, as it is uniaxially stretched while cooling by contact with the air; depending on the speed of the last pulley, it is possible to obtain fibers with different diameters. In this work, fibers with diameters ranging from 200 to 800 μm (corresponding to different draw ratios ($\mathrm{DR}\;=\;{\mathrm{diameter}}_{\mathrm{extrudate}}^{2}\;/{\;\mathrm{diameter}}_{\mathrm{fiber}}^{2}$)) were collected.

### 2.3. Characterization Techniques

Spectroscopic characterization was carried out on thin films (thickness = 50 μm) by means of attenuated total reflectance infrared spectroscopy (ATR-FTIR), using a Frontier spectrophotometer (Perkin Elmer, Waltham, MA, USA) with the following parameters: 16 scans from 4000 to 400 cm^−1^ and a 4 cm^−1^ resolution.

Rheological measurements were performed using a strain-controlled parallel plate rheometer ARES (TA Instrument, New Castle, DE, USA). Each test was carried out at 190 °C under a nitrogen atmosphere, selecting a strain amplitude value within the linear viscoelastic region of the material, in the 0.1–100 rad/s frequency range. The zero-shear viscosity was calculated by fitting the experimental data obtained through rheological tests according to Equation (1) [36,37]:

(1) $$\eta^{*}\left(\omega \right)=\frac{\eta_{0}}{{\left[\left.1+(\lambda \omega \right)\right]}^{\left(1-n\right)}}+\frac{\sigma_{0}}{\omega }$$

where η_0_ is the zero-shear viscosity, λ is the characteristic relaxation time, n is the power law index, and σ_0_ is the melt yield stress.

Rheological tests were also performed in non-isothermal elongational flow using the RheoSpin apparatus (IdeaInstr, Italy) already used for the fiber production. In particular, the melt strength, defined as the force registered by the load cell when the molten filament breaks, was recorded.

Differential scanning calorimetry (DSC) analyses were performed using a DSC Q20 (TA Instruments, New Castle, DE, USA) apparatus. The materials were subjected to the following thermal cycle: a first heating from 0 to 220 °C, a cooling run from 220 to 0 °C, and a second heating from 0 to 220 °C. All the cycles were carried out at a heating/cooling rate of 10 °C/min under a nitrogen atmosphere.

Mechanical characterization was performed through tensile tests with an Instron^®^ 5966 machine (Norwood, MA, USA) equipped with a 2 kN (error < 0.25%) loading cell. The measurement parameters for testing ISO 527-5A [38] specimens included an initial strain rate of 1 mm/min, which increased up to 10 mm/min once a deformation of 0.25% was exceeded. The tests on the fibers were performed with a crosshead speed of 20 mm/min. The average values and corresponding standard deviations of the tensile modulus, tensile strength, and elongation at break were calculated and reported. Additionally, un-notched Izod impact tests were performed using a 5.5 J pendulum impact tester HIT25P (Zwick Roell, Ars-Laquenexy, France); the energy associated with the fracture after impact was recorded.

The specimens employed for the tensile (ISO 527-5A specimens) and impact tests (dimensions: 80 mm × 12.7 mm × 3 mm) and the spectroscopic analyses were produced by compression molding through a hot-plate press Collin P200T (Collin, Maitenbeth, Germany), with the following parameters: 190 °C, 100 bar, and 3 min.

## 3. Results and Discussion

### 3.1. Effect of Degradation on the Mechanical Properties of HDPE

**Figure 1:**
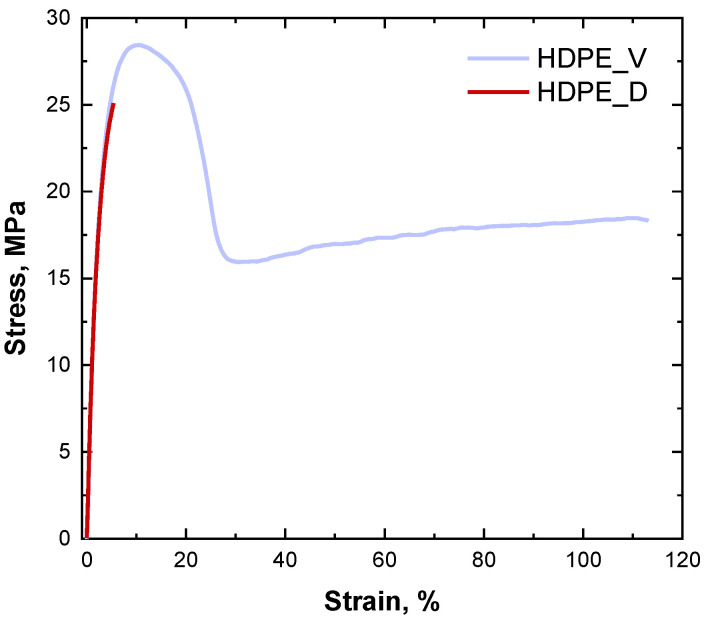
Stress–strain curves for virgin and degraded HDPE.

One of the main issues affecting the reutilization of recycled HDPE in the same application as the original material is the dramatic loss of mechanical performance, particularly in terms of ductility, compared to the virgin polymer [14,39]. To verify this feature, tensile and Izod impact tests were carried out on both degraded and virgin HDPE. Figure 1 depicts the stress–strain curves of the two samples, and Table 1 reports the averaged values of the tensile modulus and strength, elongation at break, and impact energy. An overall decrease in all the measured mechanical properties was observed when thermo-mechanical degradation occurred in HDPE. More specifically, while the tensile modulus remained almost unchanged, the elongation at break suffered a substantial reduction of 95%, passing from the virgin material to the degraded one. Likewise, the tensile strength decreased by 12% for HDPE_D. Conversely, the impact energy remained almost unchanged.

According to the literature [13,14,40], the thermo-mechanical degradation experienced by HDPE, involving a severe modification of the polymer microstructure, resulted in a severe worsening of the mechanical performances of the material. In particular, as testified by the dramatic decrease in the elongation at break, HDPE_D shows a completely brittle behavior, implying that the reutilization of this recycled material is only limited to applications with low-engineering requirements, such as the formulation of low-performance products.

### 3.2. Evolution of the HDPE Macromolecular Architecture upon Degradation and NEX Introduction

**Figure 2:**
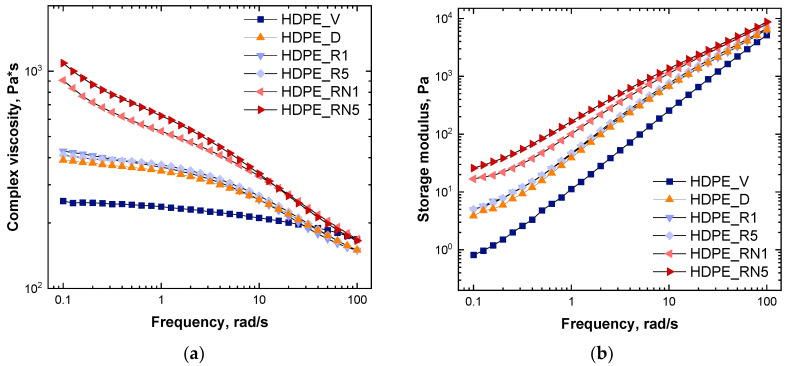
(**a**) Complex viscosity and (**b**) storage modulus as a function of the frequency for virgin (HDPE_V), degraded (HDPE_D), and recycled (HDPE_R1; HDPE_R5; HDPE_RN1; and HDPE_RN5) HDPE without and with NEX.

**Figure 3:**
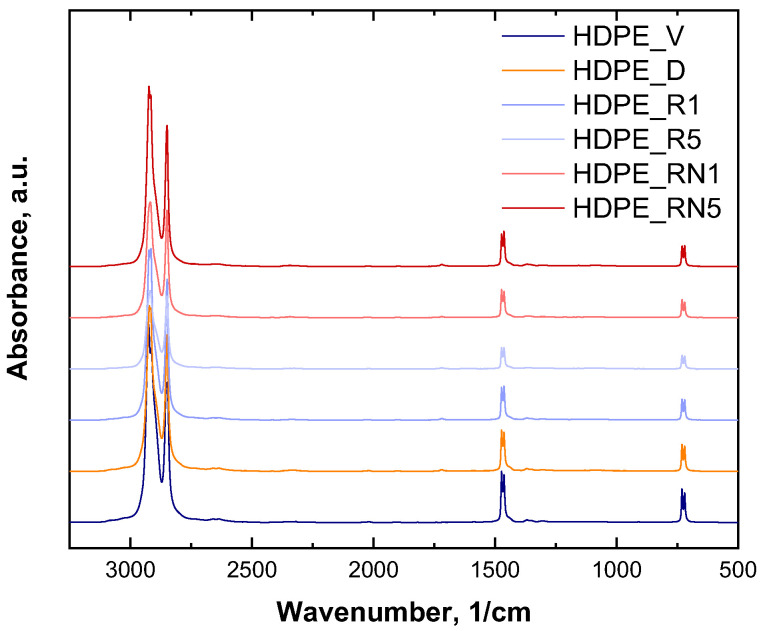
ATR-FTIR spectra for virgin (HDPE_V), degraded (HDPE_D), and recycled (HDPE_R1; HDPE_R5; HDPE_RN1; and HDPE_RN5) HDPE without and with NEX.

In order to evaluate the evolution of the HDPE macromolecular architecture induced by thermo-mechanical degradation and the possible effects of the introduction of the additive, rheological investigations were performed. Figure 2a displays the trend of the complex viscosity as a function of the frequency for all the formulated samples. Virgin HDPE (HDPE_V) features a typical Newtonian behavior at low and intermediate frequencies, with mild shear thinning when the frequency increases. The rheological behavior of HDPE_V reflects the high melt flow rate (27 g/10 min) and, thus, the low molecular weight of this polymer [41,42,43]. Concerning the degraded HDPE (HDPE_D), the complex viscosity increases in the low- and medium-frequency region with respect to HDPE_V, whereas, at higher frequencies, the curves almost overlay. The observed behavior can be attributed to the modification of the macromolecular architecture of the HDPE chains caused by thermo-mechanical degradation. In fact, as widely reported in the literature [14,20,21,22], when HDPE is reprocessed many times, different mechanisms can occur simultaneously: chain scission, the formation of long and/or short branches, and crosslinking. The higher viscosity of HDPE_D at low and intermediate frequencies compared to that of HDPE_V can thus be related to the decreased chain mobility caused by the formation of a branching structure or the introduction of some crosslinking. The inferred modification of the HDPE microstructure was further investigated through spectroscopic characterization, monitoring the extent of -CH_3_ groups on the polymer chains. As observable in Table 2 (the ATR-FTIR spectra are depicted in Figure 3, while the band assignments are reported in Table S1 in the Supplementary Materials), reporting the ratio between the intensities of the peaks related to the -CH_3_ (1368 cm^−1^) and -CH_2_ (1463 cm^−1^) groups, there is a progressive increase in this ratio when passing from virgin HDPE to its degraded counterpart. This result, coupled with the observed amplification of the non-Newtonian rheological behavior, suggests that chain scission and branching occur as competitive mechanisms during the thermo-mechanical degradation of HDPE. On the other hand, crosslinking phenomena cannot be excluded. Therefore, the macromolecules of the HDPE_D sample are characterized by a non-homogeneous architecture.

In Figure 2a, the trends in complex viscosity for recycled HDPEs are also reported. When simulating the recycling conditions for 1 min (HDPE_R1), the complex viscosity slightly increased only in the low-frequency zone compared to HDPE_D. Considering FTIR characterization (Table 2), the -CH_3_/-CH_2_ ratio decreased with respect to HDPE_D, suggesting that the crosslinking phenomenon was predominant over chain scission. When degraded HDPE was further processed for 5 min (HDPE_R5), the trend in the complex viscosity curve (Figure 2a) was very similar to that of HDPE_R1. Nevertheless, a slightly more pronounced Newtonian behavior could be observed, denoting a decrease in the molecular weight of recycled HDPE. In fact, from the FTIR analysis (Table 2), the -CH_3_/-CH_2_ ratio barely increased with increasing the processing times, indicating a growth in the number of shorter chains. Therefore, the previous characterizations suggest that, as already reported in the literature [20], chain branching is the prevailing thermo-mechanical degradation mechanism for short degradation times, while chain scission becomes predominant when HDPE is heavily degraded.

The effects of the additive were evaluated considering, as the starting material, a degraded HDPE, therefore aiming to simulate the recycling of post-consumer HDPE. To achieve this, the additive was introduced within HDPE_D, and then, the so-obtained material was further reprocessed for 1 and 5 min. Firstly, the complex viscosity displayed a significant increase in the whole tested frequency interval for the sample HDPE_RN1 (Figure 2a). Furthermore, a more pronounced shear-thinning behavior was noticed compared to HDPE_R1, and the Newtonian plateau almost disappeared. As widely reported in the literature [24,25,26,42,44,45,46,47,48], the increase in the viscosity at low frequencies and the magnified non-Newtonian characteristics could be related to the presence of long-chain branches on the polymer backbone. In fact, long-chain branches (LCBs) induce an increase in the number of chain entanglements, which prevent the mutual slipping of the macromolecules, thereby hindering their relaxation. Additionally, as reported by Stadler et al. [43], for LCB-PE samples, the typical shape of the viscosity function involves the appearance of a minimum and two distinct bends. In Figure 2a, an inflection point in the curve of HDPE_RN1 can be clearly observed. In addition, from the FTIR results reported in Table 2, an increase in the ratio between the intensities of the peaks related to methyl and methylene can be noticed for HDPE_RN1 compared to HDPE_R1, confirming the formation of LCB already inferred from the complex viscosity trend.

When the sample HDPE_RN1 was further processed, an additional increase in the complex viscosity at lower frequencies was detected, along with an amplification of the non-Newtonian features, as observable for HDPE_RN5 in Figure 2a. Once again, all the noticed modifications of the rheological response could be attributed to the presence of species increasingly hindering the relaxation of the macromolecules. The amount of -CH_3_ groups evaluated through FTIR analyses (Table 2) is diminished for HDPE_RN5 in comparison to HDPE_RN1, thereby indicating that the mean length of the side-chain branches is continuing increasing. Therefore, further processing for 5 min resulted in a higher extent of longer branches that induced an additional increase in the viscosity of the entire system. Considering that NEX is an ethylene copolymer containing hydrolysable silicon groups, according to several papers dealing with the utilization of silanes in HDPE [49,50], it can be inferred that it may act by a mechanism involving silane hydrolysis and silanol condensation reactions, leading to the formation of long-chain branching.

The latter also strongly affects the polymer melt elasticity. Looking at Figure 2b, depicting the trend in the storage modulus G′ as a function of the frequency, a progressive increase in the storage modulus in the low- and intermediate-frequency range is detected when passing from virgin HDPE (HDPE_V) to its degraded counterpart (HDPE_D). When considering the recycled HDPE (HDPE_R1, HDPE_R5), a slight increase in G′ can be observed compared to HDPE_D. On the other hand, as a result of the introduction of NEX into degraded HDPE (HDPE_RN1), the storage modulus deviates upward in the low-frequency zone, indicating a non-terminal rheological behavior. Also, in this case, the increase in G′ could be ascribed to the presence of long-chain branches and the consequent strengthening of the entanglement network [25,26,44,47,48,51]. When HDPE + NEX was further reprocessed for 5 min (HDPE_RN5), G′ displayed an additional increase at lower frequencies, indicating an even more elastic behavior due to the presence of longer side branches.

Overall, the analysis of the rheological response, coupled with the results of FTIR characterization, indicates that, when NEX is introduced within an already-degraded HDPE and the so-obtained material is further processed for different durations, the additive is able to drive the thermo-mechanical degradation reactions of HDPE towards the selective introduction of long-chain branches, promoting the achievement of a more homogeneous microstructure compared to pristine HDPE.

**Figure 4:**
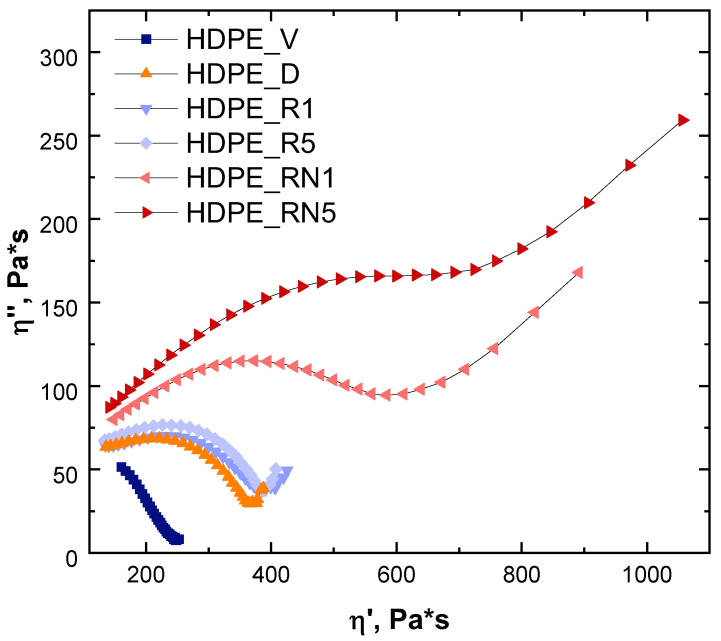
Cole–Cole plot.

In order to confirm this result, the Cole–Cole plot (displaying the imaginary component of the complex viscosity η″ as a function of the real one η′) was derived, and the obtained curves are reported in Figure 4. Typically, linear polymers show a semi-circular shape, while, for long-chain branched polymers, an upward tail (whose magnitude can qualitatively be related to the degree of long-chain branches) appears [52,53]. As shown in Figure 4, for HDPE_D, HDPE_R1, and HDPE_R5, the behavior expected for linear polymers was obtained, although the degraded and recycled materials exhibited some deviations from the semi-circular shape associable with the inferred heterogeneous microstructure encompassing some branches and crosslinking points. On the other hand, when NEX was added to the degraded HDPE (HDPE_RN1) and, even more, when the material was further processed (HDPE_RN5), a sudden increase in η″ in the high η′ region could be observed, clearly demonstrating the effect of the additive in promoting the formation of LCB, hampering the complete relaxation of the polymer macromolecules (as also demonstrated by the analysis of the relaxation spectra and the Van Gurp–Palmen plots reported in Figures S1 and S2).

It is important to highlight that all the observed alterations in the HDPE chain architecture, promoted by thermo-mechanical degradation and the incorporation of the additive, did not affect neither the crystallization and melting temperatures nor the crystallinity degree of the formulated materials, as demonstrated by the DSC analysis (see values reported in Table S2).

### 3.3. Processability Evaluations and Mechanical Performances

The previously reported characterizations revealed the ability of NEX to selectively drive the thermo-mechanical degradation reactions of HDPE towards the achievement of an LCB microstructure. As widely reported in the literature [25,28,29,54], the introduction of LCB is beneficial for obtaining the high melt strength (MS) values that are required for the successful processing of polymers through technologies (such as film blowing and fiber spinning) dominated by elongational flow. MS is a very important technological parameter in such processing operations, since it is related to the force that a stretched polymer melt can withstand before breaking. In fact, the ability of the melt to avoid deformation under its own weight is of fundamental importance when stretching or drawing is applied during the processing. In this context, if the polymer melt is unable to withstand the elongation stresses to which it is subjected at the exit of the extrusion die (e.g., tendency to sag), various issues may arise, resulting in poor-quality products or unsuccessful processing.

**Figure 5:**
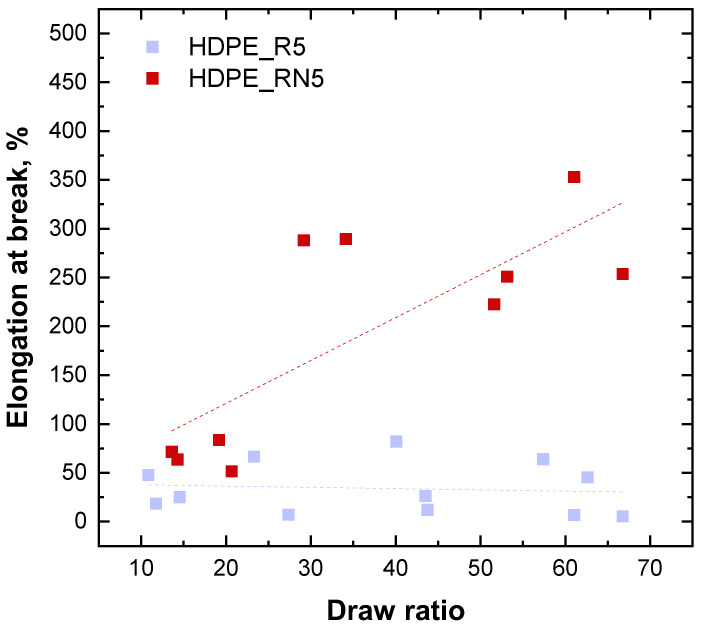
Elongation at break (%) as a function of the draw ratio for recycled HDPE without and with NEX (HDPE_R5 and HDPE_RN5).

The MS values of recycled HDPE, with and without NEX, were measured, obtaining 2.07 ± 0.27 and 4.32 ± 0.7 cN for HDPE_R5 and HDPE_RN5, respectively. Such result is quite striking and paves the way for the effective exploitation of NEX-containing recycled HDPE for further processing through technologies dominated by elongational flow. To further verify this feature, fibers of recycled HDPE without and with NEX (HDPE_R5 and HDPE_RN5) at different draw ratios were collected, and their tensile behavior was assessed. The obtained results in terms of elongation at break as a function of the draw ratio (DR) are depicted in Figure 5. As far as pristine recycled HDPE (HDPE_R5) was concerned, a slight reduction in the elongation at break with increasing the DR was observed, meaning constant or slightly reduced ductility as the diameter of the fibers decreased. However, higher values of elongation at break were obtained in the oriented samples with respect to their isotropic counterparts (see results reported in Table 1), indicating a beneficial effect of the macromolecules’ orientation. On the other hand, noteworthy results were obtained for the sample containing NEX. In fact, in this case, the fibers showed a significant growth of the elongation at break as a function of the draw ratio. As an example, at a draw ratio equal to 60, a fifty-fold increase in the elongation at break was noticed for HDPE_RN5 with respect to HDPE_R5, reaching a value of 350%.

The observed behavior could be explained considering that the presence of LCB in the sample containing NEX promoted a greater orientation capability of the macromolecular chains during continuous stretching, which, in turn, led to increased ductility [55]. Furthermore, the possible effect of LCB on the polymer crystallization kinetics cannot be neglected. In this context, we demonstrated the ability of side branches to form tie molecules in the interlamellar region between different crystalline lamellae incorporating the linear backbone, resulting in increased mechanical properties [56,57,58]. Finally, it could be inferred that the observed buildup of the elongation at break as a function of the draw ratio could have also be related to a progressive increase in the content of the amorphous phase for thinner fibers, solidifying faster than thicker ones.

To summarize, the obtained results undoubtedly demonstrate that NEX can be profitably exploited for achieving an effective upcycling of HDPE. In fact, the used additive, promoting the attainment of an LCB microstructure, makes not only recycled HDPE suitable for applications (such as fibers and films) with high-engineering requirements but, even more importantly, allows for the formulation of materials based on recovered polyethylene endowed with mechanical performances comparable to those of their virgin counterparts.

## 4. Conclusions

In this work, an effective and industrially viable route for obtaining recycled HDPE with a high melt strength was proposed.

In particular, the following key findings can be pointed out:

- The thermo-mechanical degradation experienced by HDPE during a typical mechanical recycling process involved the occurrence of different phenomena, achieving a heterogeneous microstructure that, ultimately, resulted in brittle behavior. In particular, degraded HDPE exhibited a decrease of 95% in the elongation at break and of 12% in the tensile strength compared to the virgin material. This dramatic deterioration of mechanical properties severely limits any further reutilization of the recyclate for applications with high engineering requirements.
- The introduction of Nexamite^®^ R305 was effective in selectively directing the thermo-mechanical degradation pathway of HDPE towards the achievement of a long-chain branching microstructure, which, in turn, promoted the obtainment of high melt strength values, thereby allowing the further processing of NEX-containing recycled HDPE through technologies dominated by elongational flow.
- Fibers obtained by subjecting recycled HDPE containing NEX to non-isothermal stretching exhibited a remarkably enhanced ductility compared to pristine recycled HDPE samples (elongation at break increased fifty-fold for fibers stretched at DR = 60), further demonstrating the beneficial effect of NEX in enabling high-value-added applications for recycled HDPE.

To sum up, this work clearly demonstrates the possibility of obtaining recycled HDPE potentially suitable for future applications characterized by high-engineering requirements, opening new perspectives for the effective upcycling of HDPE-based waste.

## Figures and Tables

**Table 1:** Mean values of tensile modulus and strength, elongation at break, and impact energy for virgin and degraded HDPE (HDPE_V and HDPE_D).

Sample Code	Tensile Modulus [MPa]	Elongation at Break [%]	Tensile Strength [MPa]	Impact Energy [J]
HDPE_V	1113 ± 22	114.5 ± 28.0	28.2 ± 0.4	1.04 ± 0.54
HDPE_D	1101 ± 32	5.4 ± 0.9	24.9 ± 1.2	0.93 ± 0.38

**Table 2:** Ratio between the intensities of the peaks related to -CH_3_ and -CH_2_ for virgin (HDPE_V), degraded (HDPE_D), and recycled (HDPE_R1; HDPE_R5; HDPE_RN1; and HDPE_RN5) HDPE without and with NEX.

Sample Code	-CH_3_/-CH_2_ Ratio
HDPE_V	0.037
HDPE_D	0.045
HDPE_R1	0.040
HDPE_R5	0.041
HDPE_RN1	0.044
HDPE_RN5	0.041

## Data Availability

Data are available upon request.

## Supplementary Materials

The following supporting information can be downloaded at https://www.mdpi.com/article/10.3390/polym17030382/s1: Figure S1: Stress relaxation spectra for all investigated materials; Figure S2: Van Gurp–Palmen plot for all investigated materials; Table S1: Band assignment for ATR-FTIR spectra reported in Figure 3; and Table S2: Crystallization (T_c_) and melting (T_m_) temperatures, crystallization (ΔH_c_) and melting (ΔH_m_) enthalpies, and crystallinity degree (calculated as the ratio between ΔH_m_ of the sample and the enthalpy of a 100% crystalline HDPE (293 J/g)) for all investigated materials. All the reported thermal properties have been evaluated considering the thermograms recorded in the cooling or in the second heating runs.
