# Supplementary material for: High Melt Strength Recycled High-Density Polyethylene: Evaluation of a Novel Route for Targeting the Polymer Microstructure
Source: Polymers (Basel). 2025 Jan 30;17(3):382. doi: 10.3390/polym17030382 (PMC11820430; doi:10.3390/polym17030382)
Supplement: Supplementary file 1 [file polymers-17-00382-s001.zip › polymers-3445180-supplementary.pdf]

polymers

Article

High-Melt-Strength Recycled High-Density Polyethylene: Evaluation of a Novel Route for Targeting the Polymer Microstructure

Giulia Bernagozzi [1,2], Rossella Arrigo [1,2,*] and Alberto Frache [1,2]

[1] Department of Applied Science and Technology, Politecnico di Torino, Viale Teresa Michel 5, 15121 Alessandria, Italy; giulia.bernagozzi@polito.it (G.B.); alberto.frache@polito.it (A.F.)
[2] Local INSTM Unit, 15121 Alessandria, Italy
* Correspondence: rossella.arrigo@polito.it

Supplementary Materials

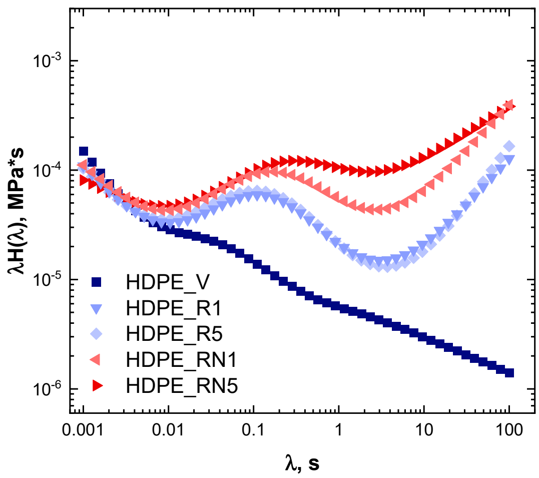

Figure S1. Stress relaxation spectra for all investigated materials.

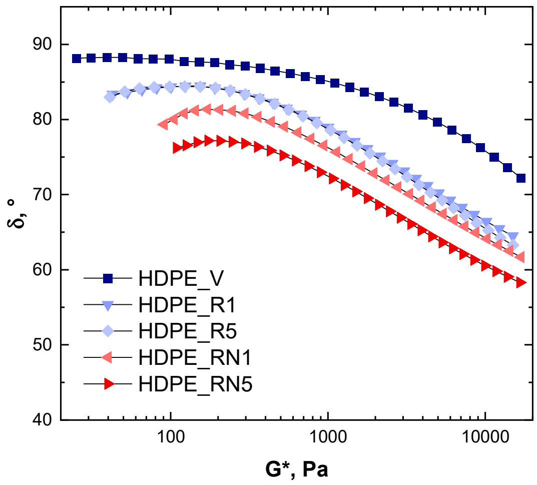

Figure S2. Van Gurp-Palmen plot for all investigated materials.

Academic Editor: Alexander Malkin

Received: 9 January 2025
Revised: 24 January 2025
Accepted: 28 January 2025
Published: 30 January 2025

Citation: Bernagozzi, G.; Arrigo, R.; Frache, A. High-Melt-Strength Recycled High-Density Polyethylene: Evaluation of a Novel Route for Targeting the Polymer Microstructure. *Polymers* **2025**, *17*, 382. https://doi.org/10.3390/polym17030382

Copyright: © 2025 by the authors. Submitted for possible open access publication under the terms and conditions of the Creative Commons Attribution (CC BY) license (https://creativecommons.org/licenses/by/4.0/).

Table S1. Band assignment for ATR-FTIR spectra reported in Figure 3.

Band [cm^{-1}]	Assignment[1]
2920	CH_2 asymmetric stretching
2850	CH_2 symmetric stretching
1473 and 1463	Bending deformation
1368	CH_3 symmetric stretching
730 and 720	Rocking deformation

[1] Gulmine, J.V.; Janissek, P.R.; Heise, H.M.; Akcelrud, L. Polyethylene characterization by FTIR. *Polym. Testing* **2002**, *21*, 557–563.

Table S2. Crystallization (T_c) and melting (T_m) temperatures, crystallization (ΔH_c) and melting (ΔH_m) enthalpies and crystallinity degree (calculated as the ratio between ΔH_m of the sample and the enthalpy of a 100 % crystalline HDPE (293 J/g)) for all investigated materials. All the reported thermal properties have been evaluated considering the thermograms recorded in the cooling or in the second heating runs.

Sample code	T_c [°C]	ΔH_c [J/g]	T_m [°C]	ΔH_m [J/g]	Crystallinity [%]
HDPE_V	115.3	207.0	131.0	213.7	73
HDPE_D	114.1	195.7	131.5	200.1	68
HDPE_R1	115.4	207.7	131.4	217.8	74
HDPE_R5	116.1	206.1	130.9	207.6	71
HDPE_RN1	116.0	204.2	130.1	209.6	71
HDPE_RN5	115.6	207.0	130.3	212.0	72
